# Laparoscopic Right Donor Nephrectomy: A Two-Center Comparative Study

**DOI:** 10.7759/cureus.59562

**Published:** 2024-05-03

**Authors:** Abdolsalam Ahmadi, Ahmed A Al Rashed, Omran Hasan, Nader Awad, Khalid Abdulaziz, Batool Turki, Sayed Dhiyaa Ebrahim, Husain Jaafar, Samer Al Geizawi

**Affiliations:** 1 Urology, Salmaniya Medical Complex, Manama, BHR; 2 Urology and Transplant, Al Saudi Hospital, Amman, JOR

**Keywords:** post-kidney transplant, live kidney donor transplantation transplant surgery, kidney transplant surgeon, urology, donor nephrectomy

## Abstract

Introduction

As the field of laparoscopic living donor nephrectomy (LLDN) has progressed over the years, there has been a growing emphasis on optimizing surgical techniques and outcomes to ensure the safety and well-being of living kidney donors. The early experiences with right LLDN, marked by challenges and concerns such as high conversion rates to open surgery and early graft loss due to technical reasons, prompted a reevaluation of the approach toward right-sided donor nephrectomies.

In this article, we aim to compare the safety and efficacy of right LLDN to left LLDN performed in our centers and to provide valuable insights that can ultimately enhance patient outcomes and ensure the well-being of living organ donors.

Methods

Between January 2018 and January 2022, we conducted 16 cases of right LLDN and compared them with 134 cases of left LLDN procedures done in the Kingdom of Bahrain and Jordan over the same time period. We analyzed differences in donor age, sex, operative time, warm ischemia time (WIT), graft function, complications, and conversion to open technique. Patient data and surgical outcomes were extracted from medical records and surgical databases.

Statistical analysis was conducted to identify significant differences between the two groups. Categorical variables such as complications and safety outcomes were compared using chi-square tests and logistic regression analysis. The primary outcomes of interest included safety metrics such as complication rates, vascular complications, graft loss, and postoperative serum creatinine levels for the recipients.

Results

Our study showed similar demographics in both groups. However, the operative time was shorter for the left LLDN, with 81 minutes compared to 96 minutes for the right. Warm ischemia times (WITs) were comparable at 4.5 minutes for the left and 5.2 minutes for the right. There was less incidence of delayed graft function on the left side (none in the left group compared to one case in the right group). Both groups had similar six-month graft function in terms of serum creatinine levels (0.98 mg/dL for the left and 1.2 mg/dL for the right), hospital stays (2.5 days for the left and 2.8 days for the right), and estimated blood loss (EBL) (90 mL for the left and 50 mL for the right). Additionally, no blood transfusions were required in either group, but there was one case of conversion to open surgery in the right LLDN group.

Conclusion

Our data confirm the safety and efficacy of the right LLDN, consistent with the current literature. This increases the cumulative evidence supporting the use of laparoscopic retrieval on the right side when indicated.

## Introduction

Historically, the evolution of laparoscopic living donor nephrectomy (LLDN) began since its inception by Clayman in 1991 [1]. Significant steps were further taken by Ratner in performing the first left LLDN in 1995, which led to the subsequent adoption of minimally invasive surgery as a standard of care in transplant centers [2]. Renal transplant discussion revolves around the selection of which kidney to retrieve with a historically clear surgeon preference for retrieving the left kidney due to its longer vein, making it more suitable for transplantation [2]. However, the need for right donor nephrectomy arises in specific situations such as better function, multiple arteries, or conditions like large cysts or stones in the contralateral kidney.

It is noted that the early challenges associated with right LLDN, including a high rate of conversions to open surgery and early graft loss, were particularly due to vascular complications as reported by Mandal, which were as high as 56% [3]. Such experiences led some surgeons to opt for the left kidney to minimize the risk of graft loss, despite the right kidney offering scientifically better options for the donor. Over time, as surgeons gained more experience with laparoscopic procedures, the results of right LLDN improved, demonstrating safety and efficacy comparable to open surgery. Furthermore, the shift toward adopting right LLDN as a viable and safe alternative to left LLDN signified a maturation in the field of minimally invasive surgery and transplant procedures. Despite the initial reservations and fears surrounding the risk of graft loss associated with right-sided procedures, advancements in surgical expertise and technology have enabled a more nuanced and refined approach to right donor nephrectomy. This was mentioned in the outcomes from the University of California, San Francisco (UCSF), which showed that right LLDN yielded similar results to the left kidney, with equivalent surgical outcomes in a series of 54 cases [4].

The comparison of outcomes between right LLDN and left LLDN represents a critical step in understanding the nuances and benefits of each approach. While traditionally left kidney is preferred for living donor surgeries, the recognition of specific scenarios where the right kidney may be a more suitable option underscores the importance of individualized patient care and surgical decision-making [5].

In this article, we aim to compare the safety and efficacy of right LLDN to left LLDN performed in our centers and to provide valuable insights that can ultimately enhance patient outcomes and ensure the well-being of living organ donors.

## Materials and methods

This research utilized a comparative retrospective design to analyze and compare outcomes between cases of right LLDN and left LLDN. In this case, data from surgeries performed between January 2018 and January 2022 was reviewed and compared. The study included cases of right LLDN and left LLDN performed by the same surgeon in multiple centers in Bahrain and Jordan during the specified time frame during which the center in Bahrain performed 27 cases whereas the center in Jordan performed 123. This criterion helps ensure consistency in surgical technique and approach across all cases being studied.

Patient data and surgical outcomes were extracted from medical records and surgical databases. Parameters such as safety metrics, complication rates, estimated blood loss (EBL), blood transfusions, length of surgery, warm ischemia time (WIT) for donor surgery, and postoperative outcomes for recipients were systematically collected and documented.

The study focused on comparing outcomes between cases of right LLDN that were identified as group 1 and left LLDN cases that were identified as group 2. By comparing outcomes between these two groups, we aimed to identify any differences in safety, complications, and other relevant parameters that may be influenced by the laterality of the donor nephrectomy. 

All cases were done with total laparoscopic technique using the upper pole first technique in all cases, which was originally described for left LLDN and kidneys were extracted through Pfannenstiel incision using manual extraction [5]. All donors underwent a full pre-operative workup including full blood count, renal function testing, and coagulation profile. A pre-operative multi-phase renal CT scan was also done for all donors to assess the anatomy and vascular structures of the donor kidney. All patients were kept Nil Per Os (NPO) at least eight hours before surgery and no other specific bowel preparation was done. All surgeries were conducted under general anesthesia performed by a consultant anesthesiologist. The surgical technique included keeping all patients in a lateral decubitus position (depending on the laterality of which the kidney is to be extracted) followed by access into the abdomen using ports and achieving pneumo-peritoneum with carbon dioxide insufflation. Additional ports were introduced under direct vision and triangulated over the target kidney. This was followed by reflection of any bowel segments and dissection of the surrounding renal fat and adhesions if present. Once the kidney is completely mobilized and blood vessels are divided, the renal hilum is located and the renal artery is clamped along with the renal vein and ureter. The kidney is retrieved through a wider incision done in the lower abdomen. WIT is measured in minutes from the moment of renal artery clamping until the kidney is removed and placed into ice for transport to the recipient. 

Statistical analysis was conducted to identify any significant differences between the two group parameters. Descriptive statistics such as mean, median, range, and standard deviation were used to summarize continuous variables like EBL, WIT, and surgical time. Categorical variables such as complications and safety outcomes were compared using chi-square tests and logistic regression analysis. The primary outcomes of interest included safety metrics such as complication rates, vascular complications, graft loss, and postoperative serum creatinine levels for the recipients. Secondary outcomes included blood transfusions and length of surgery. Furthermore, postoperative complications were classified as per the modified Clavien-Dindo classification of complications for laparoscopic nephrectomy as seen in Table 1 of the Appendix.

It was important to ensure that patient confidentiality is maintained throughout the study. Ethical approval was obtained from the relevant institutional review boards to conduct the retrospective review and analyze patient data.

## Results

During the study period, 16 right LLDN were performed and designated as group 1, compared to 134 cases of left LLDN that were performed during the same period and designated as group 2. The average donor’s age was 36 years for group 1 and 34 years for group 2, males were 31% for group 1 and 29% for group 2, indication for surgery in group 1 was multiple left renal arteries in seven cases, large right renal cysts in four cases, small right renal stones in four cases, and one case where the split function for the right kidney was 43%.

**Figure 1:**
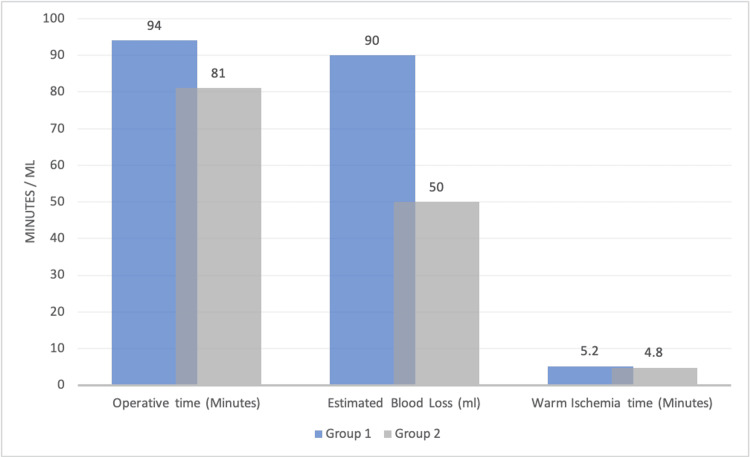
Variations in Operative Procedures Between Groups 1 and 2

In terms of surgical procedural variations, the mean length of surgery was significantly longer for group 1, 94 minutes for group 1, and 81 minutes for group 2 (P<0.0001, 95% CI), EBL was 90 in group 1 and 50 in group 2 (P=0.0128) and the WIT not considered statistically significant between both groups in which group 1 was 5.2 minutes and group 2 were 4.8 minutes (P=0.136). as demonstrated in Figure 1. 

**Figure 2:**
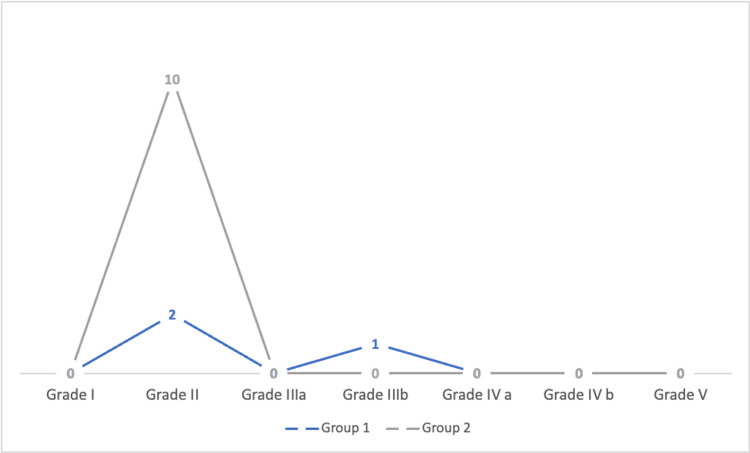
Development of Different Grades of Complications Between Both Groups As per the Modified Clavien-Dindo Classification

Furthermore, one case in group 1 required blood transfusion intra-operatively, and none in group 2. Length of stay was comparable in both groups with 2.8 days and 2.5 days (P=0.0248) for groups 1 and 2, respectively. In terms of major operative complications, one case from group 1 was converted to open surgery due to bleeding from a lumbar vein; however, none from group 2 were converted to open surgery. Aside from conversion to open surgery in one case among group 1, no other major complications were observed in both groups. In terms of common surgical complications unrelated to the procedure, one patient developed atelectasis in group 1 and seven cases in group 2 whereas paralytic ileus developed in one case in group 1 and three cases in group 2. These are illustrated in Figure 2.

In terms of recipients, one case developed delayed graft function in group 1 and none in group 2 whereas a single mortality was encountered in group 2 due to viral pneumonia, which occurred three weeks after surgery, and one graft loss due to accelerated rejection in the first six hours after surgery, which required an allograft nephrectomy in group 2. Finally, serum creatinine measured at six months was 0.98 in group 1 and 1.1 in group 2 (P=0.028), which was not statistically different between both groups.

## Discussion

The LLDN has been the standard of care in most transplant centers around the world. This procedure is favored for being less invasive, causing less pain, enabling an earlier recovery and return to normal activities, and providing better cosmetic results, all of which are encouraging for donors to proceed with the procedure [6-8]. Similar to open surgery, the left kidney is typically preferred for kidney transplantation due to the longer left renal vein, which facilitates the transplantation process. However, in certain cases, the right kidney is selected to leave the better kidney for the donor [9,10].

Initially, the experience with the right LLDN was discouraging as it was a challenging and more difficult procedure. Surgeons need to retract the liver and handle a shorter right renal vein. In some instances, the right renal artery has to be dissected under the inferior vena cava (IVC). More experience is crucial to successfully perform this procedure. Over time, right LLDN has gained acceptance and become a safe alternative to open right donor nephrectomy [10-12]. Performing right LLDN allowed for the inclusion of donors who are only suitable for right donor nephrectomy, reducing bias toward taking the left kidney when harvesting the right side might be more advantageous [13].

In our experience, we achieved comparable results between right LLDN with left LLDN. We encountered one case (6.25%) that required conversion to an open technique, which is higher than the average conversion rate of around 1.5% in more experienced centers [14]. This conversion occurred in our third case due to bleeding from a lumbar vein. The recipient experienced delayed graft function requiring dialysis in the first week after the kidney transplant but subsequently recovered with a baseline creatinine level of 1.35 mg/dL. Since then, no further cases of conversion have occurred. The procedure's safety was maintained, and there were no vascular complications on the recipient's side in our series. The duration of the surgery was measured from the insertion of the first trocar to wound closure. In our series, left LLDN was notably faster initially. However, when analyzing the data further, we observed that after the 10th right LLDN, the duration became comparable. With increasing experience, the surgical duration improves.

Moreover, kidney extraction was carried out through a Pfannenstiel incision with the peritoneum intact until extraction. WIT was similar between both groups. In fact, excluding the converted case, the WIT was even shorter for the right side. Graft function demonstrated no significant differences between the two groups, affirming the safety of performing right LLDN.

Finally, the most prevalent complication was ileus, which occurred in two cases (12.5%). Fortunately, all cases resolved spontaneously in less than seven days. While there is no definitive explanation for the prolonged ileus in the right LLDN group, it has been noted in other right LLDN series that ileus and reduced bowel function may be observed [15].

Limitations

Limitations of this study included selection bias as donors are typically excluded from surgery based on several factors including pre-existing morbidities, poor pre-operative functional status, and congenitally abnormal renal anatomy. Furthermore, given the fact that this data was collected from two independent centers from different countries, although the primary surgeon is the same, the working team and assistants were changing throughout the surgeries and this can potentially have an effect on surgical parameters such as but not limited to the total operative time.

## Conclusions

In this study, we demonstrated that the differences between right- and left-sided LLDN in terms of operative morbidity and mortality are not statistically significant. Our data also confirms the safety and efficacy of right-sided LLDN, which is consistent with the current literature. This increases the cumulative evidence supporting the use of laparoscopic retrieval on the right side when indicated.

## Appendices

**Table 1:** Modified Clavien-Dindo Classification for Laparoscopic Nephrectomy Accessed on 12 April 2024 via https://www.urologynews.uk.com/features/features/post/use-of-clavien-dindo-classification-in-urology-part-2-upper-tract

Grade	Complication
I	Hernia, pneumothorax treated conservatively, urine retention requiring catheterization
II	Atelectasis or lower respiratory tract infection, hyperkalemia, rib or intercostal pain
IIIa	Angio-embolization of bleeding vessel under local anesthesia, pneumothorax requiring chest drain
IIIb	Conversion to open surgery for reasons other than failure to progress injury to major blood vessel
IVa	Splenectomy, respiratory failure requiring ventilatory support
IVb	Multiorgan failure
V	Death
